# Unsupervised robot-assisted rehabilitation after stroke: feasibility, effect on therapy dose, and user experience

**DOI:** 10.1186/s12984-024-01347-4

**Published:** 2024-04-09

**Authors:** Giada Devittori, Daria Dinacci, Davide Romiti, Antonella Califfi, Claudio Petrillo, Paolo Rossi, Raffaele Ranzani, Roger Gassert, Olivier Lambercy

**Affiliations:** 1 Rehabilitation Engineering Laboratory, Department of Health Sciences and Technology, ETH Zurich, Switzerland; 2 Clinica Hildebrand Centro di riabilitazione Brissago, Brissago, Switzerland; 3 Future Health Technologies programme, Singapore-ETH Centre, Campus for Research Excellence and Technological Enterprise (CREATE), Singapore, Singapore

**Keywords:** Stroke, Neurorehabilitation, Robot-assisted therapy, Unsupervised rehabilitation, Self-directed rehabilitation, Neurocognitive therapy, Rehabilitation technologies

## Abstract

**Background:**

Unsupervised robot-assisted rehabilitation is a promising approach to increase the dose of therapy after stroke, which may help promote sensorimotor recovery without requiring significant additional resources and manpower. However, the unsupervised use of robotic technologies is not yet a standard, as rehabilitation robots often show low usability or are considered unsafe to be used by patients independently. In this paper we explore the feasibility of unsupervised therapy with an upper limb rehabilitation robot in a clinical setting, evaluate the effect on the overall therapy dose, and assess user experience during unsupervised use of the robot and its usability.

**Methods:**

Subacute stroke patients underwent a four-week protocol composed of daily 45 min-sessions of robot-assisted therapy. The first week consisted of supervised therapy, where a therapist explained how to interact with the device. The second week was minimally supervised, i.e., the therapist was present but intervened only if needed. After this phase, if participants learnt how to use the device, they proceeded to two weeks of fully unsupervised training. Feasibility, dose of robot-assisted therapy achieved during unsupervised use, user experience, and usability of the device were evaluated. Questionnaires to evaluate usability and user experience were performed after the minimally supervised week and at the end of the study, to evaluate the impact of therapists’ absence.

**Results:**

Unsupervised robot-assisted therapy was found to be feasible, as 12 out of the 13 recruited participants could progress to unsupervised training. During the two weeks of unsupervised therapy participants on average performed an additional 360 min of robot-assisted rehabilitation. Participants were satisfied with the device usability (mean System Usability Scale scores > 79), and no adverse events or device deficiencies occurred.

**Conclusions:**

We demonstrated that unsupervised robot-assisted therapy in a clinical setting with an actuated device for the upper limb was feasible and can lead to a meaningful increase in therapy dose. These results support the application of unsupervised robot-assisted therapy as a complement to usual care in clinical settings and pave the way to its application in home settings.

**Trial registration:**

Registered on 13.05.2020 on clinicaltrials.gov (NCT04388891).

**Supplementary Information:**

The online version contains supplementary material available at 10.1186/s12984-024-01347-4.

## Background

Stroke often leads to long-term upper limb impairments [1], which may limit stroke survivors during activities of daily living and negatively impact their independence and quality of life.

Therapy can promote recovery and there is growing evidence for therapy dose being a relevant factor influencing sensorimotor recovery, with a higher dose of upper limb therapy contributing to better functional outcomes, even in the chronic phase of stroke [2–4]. Current rehabilitation programs are mainly based on supervised one-to-one therapy sessions. Thus, an increase in the dose of therapy for stroke patients, without decreasing its quality, is strongly limited by factors such as low therapist to patient ratios [5] and high rehabilitation-related costs of supervised therapy.

Unsupervised robot-assisted rehabilitation, namely patients training with rehabilitation robots without the supervision or intervention of any external person, bears the potential for increasing therapy dose without significantly weighing on the healthcare system [6]. Rehabilitation robots, here intended as actuated devices that are computer controlled, can actively support movements. Compared to sensor- or VR-based technologies, this allows them to train on a wider range of impairment and makes them more suitable for patients with more severe motor deficits who require active assistance during movements. Furthermore, rehabilitation robots can objectively measure metrics related to sensorimotor ability and reproduce a variety of tasks normally performed by therapists. These features would allow rehabilitation robots to monitor progress throughout a therapy program, adapt the exercises accordingly, as well as provide feedback on performance and progress.

Unfortunately, rehabilitation robots are often complicated to setup and use (i.e., they have low usability), lack the features that would allow automatic adaptation of therapy parameters to the patient state, or might raise concerns in terms of safety. These factors may negatively affect patients’ motivation to train as well as compliance to robot-assisted therapy, and are some of the reasons why a completely unsupervised use of rehabilitation robots has not yet become a standard. The external supervision still required for active devices can take the form of therapists minimally supervising therapy sessions [7], performing remote monitoring [8] or regular meetings to adjust the exercises [9], or caregivers being present to help with using the robot [10]. Therefore, a suitable device and therapy approach to fully exploit the promise of rehabilitation robots to increasing therapy dose without adding significant burden on healthcare practitioners or other external persons remains to be explored.

In this paper, we report on a pilot study investigating the feasibility of unsupervised robot-assisted therapy with a rehabilitation robot for upper limb sensorimotor training, namely the ReHapticKnob [11, 12], in a clinical setting. Subacute stroke patients underwent a four-week protocol, where they progressively transitioned from supervised (i.e., therapist present) to unsupervised (i.e., independent) use of the rehabilitation robot. In that last phase, the robot was freely accessible and no external intervention nor supervision occurred to help interacting with the device or monitor and adapt therapy. The primary goals of this study were to (i) evaluate the feasibility of this approach, (ii) investigate the effect of unsupervised robot-assisted rehabilitation on the overall therapy dose, as well as (iii) assess user experience during unsupervised use of the robot and its usability. The secondary objective was to identify factors (e.g., age, cognitive scores) potentially influencing the feasibility and the achieved dose of unsupervised rehabilitation.

This work is important as it may help establish unsupervised robot-assisted therapy as a feasible and safe method to increase therapy dose with minimal additional workload on any external person, therefore maximizing the efficiency of robot-assisted rehabilitation and opening the door for its application in home settings.

## Methods

### The ReHapticKnob

**Fig. 1:**
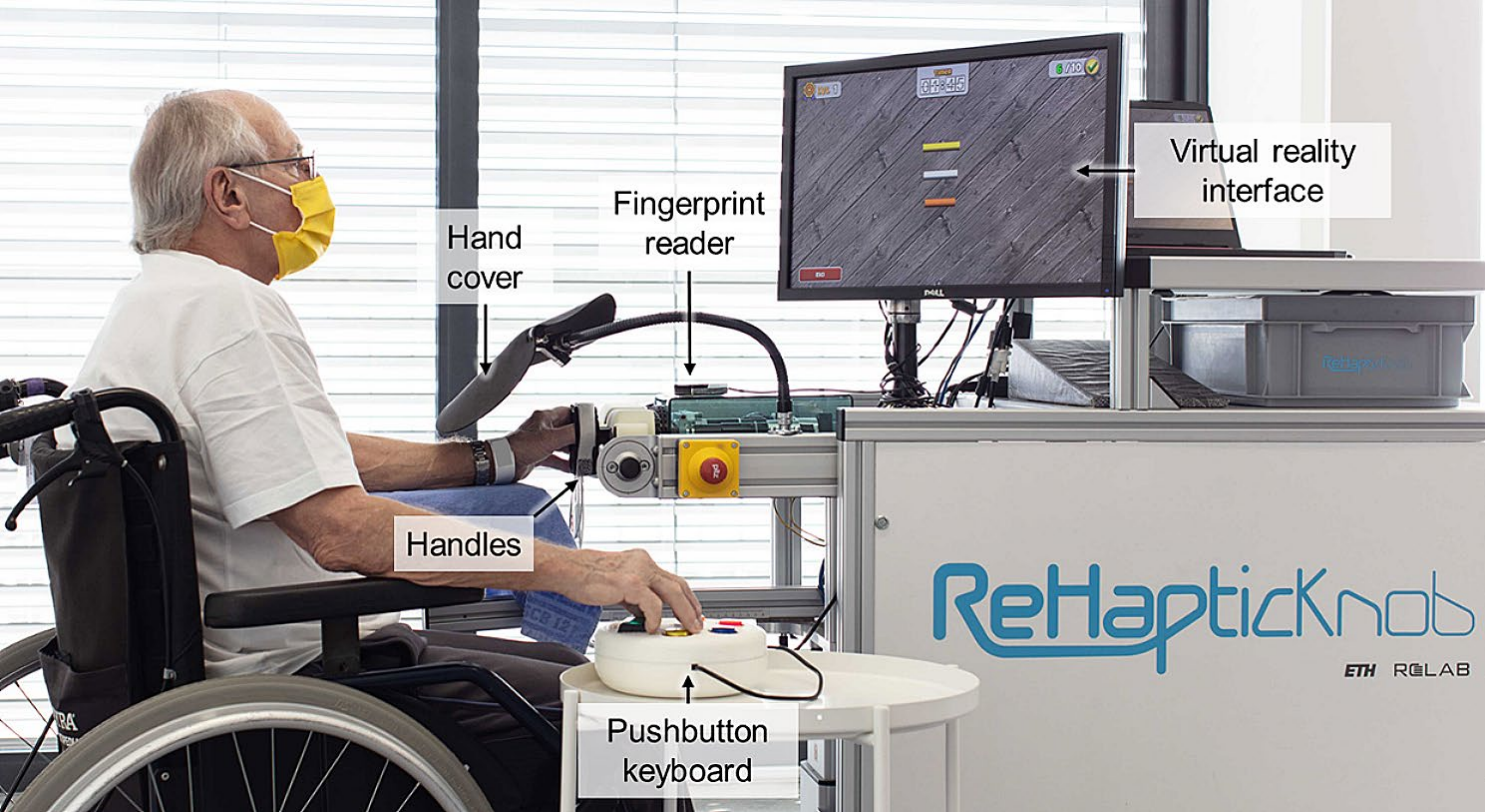
Participant performing a therapy exercise with the ReHapticKnob. To train with the device, participants need to fix their fingers to the handles with Velcro straps and log in to their therapy account with the fingerprint reader. A pushbutton keyboard is used to interact with the device and the virtual environment displayed on the screen. The view of the hand (visual feedback) is blocked by the hand cover, as the exercises require users to focus on the sensory feedback from the affected hand to solve the different tasks

The ReHapticKnob [11, 12] is an end-effector device for sensorimotor rehabilitation of the hand and forearm after stroke (Fig. 1). In previous clinical trials, therapy assisted by the ReHapticKnob and supervised by a therapist was shown to be equivalent (i.e., non-inferior) to carefully dose-matched conventional therapy [13].

A set of seven therapy exercises implemented on this device are based on the neurocognitive therapy concept, which focuses on the integration of motor, sensory, and cognitive functions when performing a task [12, 14]. The exercises focus on the passive or active training of grasping or forearm pronosupination and target subjects with different levels of impairments. The tasks subjects must perform during the exercises include, for example, interacting with virtual objects with different mechanical properties (e.g., different length or different stiffness), memorizing these, and later identifying them based exclusively on the somatosensory input from the impaired limb. In this case, a correct answer corresponds to the correct identification of the object. More details on all tasks and exercises can be found in [12, 14]. Furthermore, each exercise follows an assessment-driven concept, meaning that the initial difficulty level is tailored to the results of specific assessments performed with the ReHapticKnob before the start of the training (for more details see [15]).

To address the challenges raised by unsupervised use, a major focus was placed on improving the usability of the robot, including pilot evaluations with stroke patients and therapists [12]. For example, the graphical user interface was redesigned to be more intuitive and pleasant. Furthermore, clinically-inspired algorithms based on the action and decision process usually performed by therapists in a supervised session were implemented, with the objective of automatically monitoring, controlling, and adapting the content of therapy sessions [16, 17]. For instance, these algorithms automatically adapt the difficulty of an exercise or add a new, more challenging, exercise based on the performance, and provide feedback to guide users through the therapy sessions. Generally, they further decrease the number of actions that need to be learned to interact with the device, thereby increasing usability, and avoiding the need for a therapist to monitor and adjust therapy content over an extended period of time.

### Study protocol

This pilot study was approved by the Swiss notified body regulating the use of medical devices (Swissmedic 102681300) and the cantonal ethics commission of Ticino (CE TI 3577). A detailed description of the study protocol is provided in [18].

In short, a sample of 13 participants was chosen, which was considered large enough for a feasibility study while also taking into account a similar group size and drop-out rate (around 20%) compared to our previous studies with the ReHapticKnob [13]. Participants were recruited from the stroke inpatients of the Clinica Hildebrand Centro di riabilitazione Brissago. Inclusion criteria were age between 18 and 90 years old, inclusion within 6 weeks from stroke onset, pre-stroke modified Rankin score [19] ≤ 1, National Institutes of Health Stroke Scale (NIHSS) [20]  ≥ 1 in at least one of the items regarding motor or sensory function and ataxia, and signed informed consent form. Exclusion criteria were moderate to severe aphasia (Goodglass-Kaplan’s scale [21] < 3), moderate to severe cognitive deficits (levels of cognitive functioning-revised (LCF-R) [22] < 8), functional impairment of the upper limb due to other pathologies, severe pain in the affected arm (visual analogue scale for pain (VASp) ≥ 5), other pathologies possibly interfering with the study, pacemakers and other active implants, and modified Ashworth Scale [23] > 2 for one or more of the following muscles: shoulder adductors, forearm pronator and supinator, flexors and extensors of elbow, wrist, and fingers.

In order to teach participants to confidently use the device in an unsupervised manner and reduce the risk for adverse events, we specifically designed a systematic protocol for the progressive transition from supervised to unsupervised use of the device [18].

For each participant, the study protocol lasted four weeks. The first week consisted of 5 sessions of 45 min of supervised therapy, where a therapist was present to explain how to use the device and perform the different exercises. The second week consisted of 5 sessions of 45 min of minimally supervised therapy, i.e., participants tried to perform the therapy session independently, but a therapist was still in the room. Despite being present, in this phase the therapist remained in the background and intervened solely upon participant’s request or when necessary (e.g., for safety reasons). At the end of the minimally supervised week, the therapist evaluated the participant’s readiness to continue to the unsupervised phase as well as independence with respect to mobility (e.g., ability to independently access the device) with a custom-made checklist. If participants reached all the goals on the checklist, they proceeded to two weeks of fully unsupervised training. In the unsupervised phase, the device was kept turned on in a freely accessible room in the clinic, and participants could train during a 45-minute timeslot indicated on their daily schedule (business days only), as well as in their free time, evenings, and weekends (the latter not indicated on their schedule). Although for business days a timeslot was booked on their daily schedule to avoid interfering with the conventional therapy plan and to ensure device availability, it is important to note that participants were clearly told that therapy with the ReHapticKnob was voluntary, and no recommendations were given on the daily therapy dose to achieve. Access to the robot was also not monitored nor directly encouraged during the unsupervised phase. If, at the end of the minimally supervised week, a participant was deemed not ready to safely train unsupervised, an additional week of minimally supervised therapy was added. At the end of this second minimally supervised week, the checklist was repeated and if the requirements were met, the participant could train unsupervised for the final week. If not, a third week of minimally supervised therapy was performed.

During each phase, all the robot-assisted therapy sessions were an addition to the conventional therapy plan of the participants (usual care). Participants with very limited mobility (i.e., unable to move on their own from their room to the various therapy stations) were accompanied to the ReHapticKnob upon request by clinical staff dedicated to escorting patients to the various rooms, as for any other conventional therapy. These staff were not trained in the use of the ReHapticKnob, so while they could assist patients in positioning themselves in front of the robot, they were not allowed to help them in interacting with it.

At the beginning and at the end of the study, clinical assessments were performed. Questionnaires to evaluate usability and user experience were performed after the first week of minimally supervised therapy (Usability 1) and at the end of the study (Usability 2), to evaluate the change in perceived usability due to therapists’ absence during the robot-assisted therapy sessions. User experience was further evaluated at the end of each therapy session with the ReHapticKnob by automatically presenting the question “How was your therapy session today?”, to which subjects could answer with a 5-point Visual Analogue Scale represented by different emoticons (VAS – Smiles).

### Primary outcome measures

Feasibility of the proposed protocol was measured as the number of subjects who could proceed to the unsupervised phase, safety of use (i.e., number of adverse events and device deficiencies), and attendance during the unsupervised phase. Attendance is here defined as the percentage of days where the participant trained with the ReHapticKnob at least once (detected by the login in the therapy account) out of the total number of offered days for unsupervised therapy (i.e., 14 or 7).

A further outcome was the dose of unsupervised robot-assisted therapy, measured as therapy duration in minutes per day and total minutes over two complete weeks of unsupervised therapy, number of task repetitions, and percentage increase in physical therapy time due to the robot-assisted therapy with respect to the conventional physical therapy time (i.e., upper limb and lower limb physio- and occupational therapy) during the unsupervised phase. The latter metric reflects the increase in therapy dose that could be achieved with minimal use of the clinical resources. Conventional physical therapy time was precisely calculated for each participant based on their conventional therapy plan provided by the clinical administration.

An additional primary outcome was the change in usability and user experience between Usability 1 and Usability 2. Performed usability questionnaires included the System Usability Scale (SUS) [24], the raw Task Load Index (TLX) [25], and the Post-Study System Usability Questionnaire (PSSUQ) [26]. User experience was characterized with the net promoter score (NPS) [27], and the customer satisfaction score (CSAT). In this case, the NPS reflected the probability (on a scale from 0 to 10) with which a participant would recommend therapy with the ReHapticKnob to another patient. Participants are divided into promoters (score 9 or 10), passively satisfied (score 7 or 8), and detractors (score < 7). The final score is given by the subtraction of the percentage number of detractors from the percentage of promoters, with higher values corresponding to a higher ratio of promoter to detractors. For the CSAT, participants had to rate their level of satisfaction with the therapy with the ReHapticKnob on a 5-point scale ranging from “very unsatisfied” (i.e., 1) to “very satisfied”.

The difference between VAS – Smiles ratings given in the three phases was used to further investigate how user experience changed depending on therapist’s presence. The results of the custom-made checklist were also used to identify aspects of the device possibly requiring improvement.

### Secondary outcome measures

The difference in the content of the therapy sessions between the three phases (supervised, minimally supervised, and unsupervised) was evaluated to investigate whether, in the absence of the therapist, participants really engaged in the exercises of their personalised therapy plan and did not, for example, simply start the exercises without performing them. Thus, for all three phases we expected no significant difference in therapy content. The metrics used to assess therapy content were intensity (i.e., number of task repetitions per minute), task performance (i.e., correct responses out of the total number of repetitions), and ratio of effective therapy time (i.e., net therapy time without breaks) to total duration of a therapy session.

An additional secondary outcome was functional recovery, calculated as the difference between final and baseline scores for the clinical assessments. The assessments performed at both time points were the Fugl-Meyer Assessment of Upper Extremities (FMA-UE) [28], ABILHAND [29], Box and Block test (BBT) [30], Motor Evaluation Scale for Upper Extremities in Stroke Patients (MESUPES) [31], and modified Ashworth Scale (mAS) [23].

Furthermore, parameters possibly influencing unsupervised therapy dose or attendance were investigated. These parameters included age, baseline clinical assessments scores, and dose of conventional therapy in minutes during the unsupervised phase. The impact of cognitive deficits and of the level of independence with respect to mobility, measured with the Barthel Index and custom questions, on the ability to proceed to the unsupervised phase was also investigated.

### Data analysis

Descriptive statistics (mean and range (min-max) or boxplots) were computed for study population, attendance and therapy dose in the unsupervised phase, user experience, platform usability, assessments scores, and functional recovery. Therapy dose as total minutes over the unsupervised phase was calculated only for subjects who achieved two complete weeks of unsupervised therapy.

The paired samples Wilcoxon test was used to compare the data collected during Usability 1 and Usability 2. Subjects who did not perform unsupervised therapy and subjects who did not complete both usability sessions were excluded from this analysis.

The Friedman test was performed to compare therapy content (i.e., mean intensity, performance, and effective therapy time for each subject) and mean VAS – Smiles ratings between the three phases. The Wilcoxon signed rank test with Bonferroni correction was used for post hoc analysis. Subjects who did not reach the unsupervised phase were excluded from the analysis.

Linear fixed-effects models were computed to investigate parameters possibly influencing the achieved total dose of unsupervised therapy and attendance in the unsupervised phase. The parameters included were age, dose of interdisciplinary conventional therapy in minutes (as an estimate of fatigue), and baseline scores for the clinical assessments (i.e., ABILHAND, BBT, FMA-UE, MESUPES). Given the limited dataset, we had to restrict the number of independent variables of the model. Therefore, we selected the parameters that are known soon after admission to the clinic and that in the future could potentially be early predictors of which patients are good candidates for unsupervised therapy. Subject who did not perform two complete weeks of unsupervised therapy were excluded from this analysis. Significance level was set to 0.05.

## Results

Thirteen subjects were recruited for this study (Table 1). Of these, two did not fully complete the study due to early discharge from the clinic but were still considered for analysis. Baseline clinical assessment scores are reported as part of Table 4.

**Table 1:** Subjects’ characteristics

	Mean	Range	Ratio
Age (years)	65.9	49–77	
Gender (Female:Male)			3:10
Time after stroke (days)	14.7	7–33	
Stroke type (Ischemic:Haemorrhagic)			10:3
Impaired side (Left:Right)			7:6
Goodglass-Kaplan Scale (5 = minimum impairment)	4.4	3–5	
LCF-R (10 = minimum impairment)	9.4	8–10	

### Feasibility

Twelve out of the 13 participants could progress to unsupervised training with the ReHapticKnob, while one (Participant 4) did three weeks of minimally supervised therapy. Of these twelve, one subject needed two weeks of minimally supervised therapy before going to the unsupervised phase.

All subjects attended all the sessions of the supervised and minimally supervised phases (100%). Mean attendance during the unsupervised phase was 77.0% (range: 64.3–90.9%), and six subjects trained at least twice during the two weekends included in the unsupervised phase. Regarding safety of use, no adverse events or device deficiencies occurred.

### Dose of unsupervised robot-assisted therapy

**Fig. 2:**
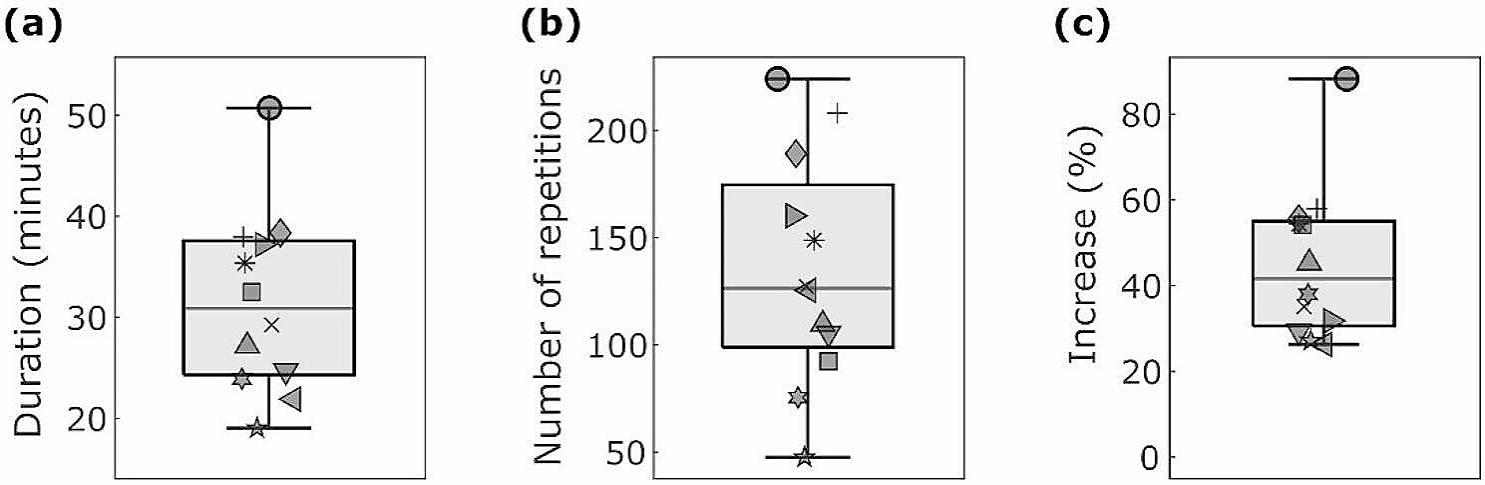
Boxplots of the therapy dose achieved during the unsupervised phase. (a) Mean daily therapy duration in minutes and (b) mean number of daily task repetitions for each subject and only for the days when they trained with the ReHapticKnob. (c) Percentage increase in therapy time due to the robot-assisted therapy with respect to the conventional physical therapy program only (i.e., physio- and occupational therapy). Different markers represent the different participants

The mean daily therapy dose in minutes and in number of repetitions, as well as the percentage increase in physical therapy time through the unsupervised training are shown in Fig. 2. The mean total robotic therapy time over two complete weeks of unsupervised therapy was 360.0 min (range: 197.2–608.7 min).

According to the linear model, the achieved total dose of unsupervised therapy seemed to be significantly affected by both age (t = -5.1, p-value = 0.036) and baseline FMA-UE score (t = -4.4, p-value = 0.049), with increasing age and FMA-UE score leading to slightly decreased dose of unsupervised therapy (see Additional file 1). Attendance was not significantly affected by any of the parameters tested (see Additional file 2).

## Usability and user experience

Usability outcomes are reported in Table 2. No significant difference was found between the Usability 1 and Usability 2 sessions. The net promoter score was 27.3% (out of 100) for both Usability 1 (6 promoters, 3 detractors) and Usability 2 (5 promoters, 2 detractors). The customer satisfaction score was 81.8% for Usability 1 and 80.0% for Usability 2. Regarding the VAS – Smiles ratings, no significant difference was found between the three different phases. Mean VAS – Smiles ratings was 3.8 (out of 5) for the supervised and minimally supervised phase, and 3.5 for the unsupervised phase.

**Table 2:** Results of the descriptive statistics and paired samples Wilcoxon test for the usability metrics

Outcome measure	Usability 1		Usability 2		Difference		Wilcoxon test: p-value
	Mean	Range	Mean	Range	Mean	Range	
SUS	79.8	55.0- 100.0	85.0	35.0-100.0	5.2	-25.0-30.0	ns
TLX	40.3	15.0–60.0	39.2	30.0-53.3	-1.1	-21.7-21.7	ns
PSSUQ	2.0	1.1–2.8	2.0	1.1–4.9	0.0	-1.2-2.5	ns

SUS: System Usability Scale. TLX: raw Task Load Index. PSSUQ: Post-Study System Usability Questionnaire. ns: non-significant

The participant who could not train unsupervised (Participant 4) did not reach the goal of completing all minimally supervised therapy sessions without the need to call for help for relevant reasons, as for instance he was often applying too much force to the handles and the device then went into safety mode, blocking the handles and requiring the intervention of an external person to continue with the exercises.

The subject who needed one additional week of minimally supervised therapy did not initially reach the goal of placing the hand on the robot in the correct way, as motor impairments in the arm not trained with the device made it difficult to independently fasten the Velcro straps.

### Secondary outcomes

Outcomes related to the content of robot-assisted therapy in the three phases of the study protocol are reported in Table 3. Intensity was the only metric significantly different between the three phases, with post hoc analysis showing that intensity in the minimally supervised phase was significantly higher than in the supervised phase (p-value with Bonferroni correction: 0.015).

**Table 3:** Therapy content for the supervised, minimally supervised, and unsupervised phases

Outcome measure	Supervised		Minimally supervised		Unsupervised		Friedman test: p-value
	Mean	Range	Mean	Range	Mean	Range	
Ratio effective to total therapy time	0.8	0.7–0.9	0.8	0.7–0.9	0.8	0.5–0.9	ns
Intensity (repetitions/ minute)	3.6	2.5–5.1	4.1	3.2–6.1	4.0	1.9–5.2	0.0052
Task performance (%)	64.9	46.9–75.9	69.3	60.0- 78.6	70.7	60.6–78.2	ns

The p-values of the Friedman test used to compare the results between the different phases are reported. ns: non-significant

Table 4 summarizes the results related to functional outcomes.

**Table 4:** Descriptive statistics for the clinical and robotic assessments

Outcome measure	Baseline		Final		Difference	
	Mean	Range	Mean	Range	Mean	Range
FMA-UE	50.6	22.0–63.0	60.3	39.0–66.0	9.7	3.0–26.0
ABILHAND	20.7	7.0–36.0	35.1	21.0–46.0	14.2	4.0–32.0
BBT	24.6	0.0–43.0	36.9	13.0–65.0	12.2	0.0–36.0
MESUPES	44.1	11.0–56.0	55.5	40.0–58.0	11.3	2.0–29.0

FMA-UE: Fugl-Meyer Assessment of Upper Extremities. BBT: Box and Block test. MESUPES: Motor Evaluation Scale for Upper Extremities in Stroke Patients

Four subjects had very low independence with respect to mobility, meaning that they had a Barthel Index of 0 (immobile), were in a wheelchair, and had to be accompanied to all the therapy sessions with the ReHapticKnob by the dedicated clinical staff. Of these, two could train unsupervised for two weeks, one for one week, and one (Participant 4) did not reach the unsupervised phase.

The participant who could not train unsupervised (Participant 4) obtained the lowest score regarding cognitive function (LCF-R = 8). The subject with the worst score for aphasia (Goodglass-Kaplan = 3) was able to train two weeks unsupervised.

## Discussion

The main goals of this study were to assess feasibility of unsupervised robot-assisted therapy in a clinical setting, evaluate the potential increase in therapy dose that could be achieved with this therapy modality, and evaluate usability of the tested robot (ReHapticKnob) as well as user experience during its unsupervised use. The secondary goal was to identify potential factors influencing the feasibility or achieved dose of unsupervised robot-assisted rehabilitation.

The results presented here demonstrate that unsupervised therapy with the actuated device ReHapticKnob in a clinical setting is feasible and allows to increase the dose of therapy.

Contrary to other studies, where for example family members or caregivers were involved [10, 32], no external intervention was provided during the unsupervised phase of our study to help participants interact with the robot or to monitor or adjust therapy parameters. The only intervention performed by the clinical staff during the unsupervised phase was turning the robot on and off in the mornings and evenings and, as for conventional therapies, accompanying participants with very limited mobility to the device, eventually helping them to position the wheelchair but in no way helping them to place their hand on the device or interact with it. This implied minimal use of clinical resources, as the clinic has staff dedicated precisely to transporting patients from one therapy site to another. No further assistance was provided. The use of these resources was therefore related to the patient’s condition and not to the device and intervention.

### Unsupervised robot-assisted rehabilitation was feasible

Unsupervised therapy with the ReHapticKnob was found to be generally feasible in our patient population, as 12 out of 13 participants could train unsupervised and no adverse events or device deficiencies occurred.

The mean attendance found in this study (76.4%) is higher than the one reported in other studies in which rehabilitation technologies were used with minimal or no supervision. For instance, in [33], subjects with chronic stroke on average trained with a sensor-based system without supervision for 26.5 days out of 42 (63%), while in [34] subjects on average performed 55% of the sessions proposed over four weeks of semi-autonomous training at the clinic with a passive device (ArmeoSpring). The higher attendance observed in our study might be due to ReHapticKnob being an end-effector device, which makes it easy and quick to set up, and to the efforts made to first familiarize participants to the use of the device during the supervised phase [18]. Furthermore, the fact that study participants had a suggested timeslot for training with the device printed on their daily therapy program might have promoted attendance. Also worth noting is the fact that without considering weekends, the average attendance in our study was 88.7%. During the weekends, participants did not receive any document with their daily program, as no conventional therapy was performed. This, together with the fact that during weekends patients are used to not having therapies and may occupy their time with other, non-therapy related activities (one participant even went home for the weekend once during the unsupervised phase), may explain the lower attendance during weekends.

We could have chosen to exclude patients with severe motor impairments, and therefore very limited mobility, but since our device also offers therapy exercises suitable for patients with severe hemiparesis [15], we decided to investigate a population with a wide range of impairment level (range for baseline FMA-UE and BBT were 22–63 and 0–43, respectively). The majority of these patients could use the device without supervision, suggesting that our approach is feasible independent of the impairment level. As outcomes among subjects with low independence regarding mobility varied in terms of ability to train without supervision, it can be concluded that mobility was not a limiting factor. However, mobility might generally be limiting in terms of attendance and unsupervised therapy dose, as those participants depended on the clinical staff to accompany them to the device, and the staff is reduced during weekends. Having such a device in the participant’s room or, at a later stage, directly at home, could help overcome this issue.

Age did not seem to play a major role in the ability to train unsupervised, as attendance was not affected by it and the three oldest subjects (77 years old) had different outcomes in terms of number of unsupervised weeks. Although we excluded patients with moderate to severe cognitive impairments, the subject with the lowest LCF-R score (i.e., 8) could not train unsupervised, so the threshold for the exclusion criteria might have been too low. However, since only one subject with this score was recruited, it is difficult to draw a definitive conclusion.

As expected, the content of the therapy sessions (i.e., the ratio of effective to total therapy time, intensity, and performance) was similar across the three phases, suggesting that the therapist’s absence had no negative influence on participants’ engagement or ability to perform the exercises. The significant increase in intensity between the supervised and minimally supervised phases could be due to a learning effect, which may have led to patients needing fewer breaks (e.g., for explanations or interactions with the therapist) and being more efficient. The performance results, which were similar between the three phases, support the idea that higher intensity is not due to patients doing the exercises hastily or without paying attention.

### Unsupervised robot-assisted therapy allowed to increase therapy dose

Over two weeks, participants performed around 6 h of fully unsupervised robot-assisted therapy on average. High-intensity upper limb therapy programs that have been shown to have benefits for patients involve higher doses of therapy, e.g., 25–30 h per week [3, 4]. However, participants in our study were inpatients with an already extensive therapy schedule, not chronic patients as in the previously mentioned studies. Furthermore, we did not provide specific recommendations on how much to train with the device, in an attempt to evaluate the real willingness to engage in unsupervised therapy. It could be expected that the achieved dose of unsupervised robot-assisted therapy could be further increased by setting daily targets and providing feedback or reminders when targets are not reached [35].

The average daily dose of unsupervised therapy in minutes achieved by these participants is comparable to other studies investigating the use of rehabilitation technologies with minimal or no supervision. For instance an average of around 31 [9], 33 [34], or 29 [36] min/day were reported, for study protocols of different lengths. The average total dose achieved during the two unsupervised weeks (360 min, i.e., 180 min/week on average) was slightly higher than the average weekly dose of home training with the SCRIPT passive device registered over 6 weeks in [37], which was of 105 min/week. The large variability observed in the unsupervised therapy dose between participants also compares to other studies [9, 33, 38]. Although the sessions with the robot were planned to last 45 min, the actual duration recorded by the robot was typically shorter, even during the phases when the therapist was present. From what was observed during the study, this was mainly due to fatigue that led patients to stop the exercises a little earlier, and to a lesser extent also to the time required for patients to move from one therapy to another and for set up (e.g., positioning and securing the hand at the beginning, disinfecting the device at the end). In the future, it might be recommended to train with the robot for less consecutive time but multiple times a day.

The number of daily repetitions achieved in the unsupervised phase is higher than the mean typically reported for conventional therapy [39, 40], which underlines that the additional dose that could be achieved via unsupervised therapy is meaningful. This is in line with the general trend of technologies allowing more intensive training [41].

As this protocol was designed to have a similar length and structure to our previous study [13], a randomized controlled trial analysing supervised use of the ReHapticKnob and usual care (same clinic and usual care program), a careful comparison in terms of clinical outcomes is possible. In the present study, the main differences were that robot-assisted therapy (in all three phases) was an addition to usual care and not a substitution of conventional therapy sessions, and that the measured number of repetitions was found to be higher, as dose was not limited. Despite the similar baseline characteristics with respect to age (Wilcoxon rank sum test: p-value = 0.29) and FMA-UE score (Wilcoxon rank sum test: p-value = 0.61) of the participants, the present study providing access to additional therapy with the device led to a larger decrease in upper limb impairment (mean increase in FMA-UE: +6.75 for both groups together in [13], while + 9.67 in the hereby described study, i.e., + 43%). This would support the assumption that increasing therapy dose has the potential to support further improvements.

Despite not having an influence on the ability to train unsupervised, age significantly impacted the dose of unsupervised therapy. This might be related to older persons experiencing higher levels of mental fatigue [42], which may in turn have a negative impact on physical activity levels [43] and, in our case, on the dose of therapy with the ReHapticKnob, which involves both physical and mental aspects. Dose was also significantly influenced by baseline FMA-UE scores, with the output of the linear regression model suggesting that persons with a higher impairment level invested more time in the unsupervised therapy.

### Usability and user experience were generally good and were not significantly affected by the absence of the therapist

The difference between the outcome measures recorded in Usability 1 and 2 is not significant, indicating that the therapist’s absence did not lead to a decrease in usability and user experience and that the familiarization phase was effective in making participants comfortable with using the ReHapticKnob independently.

According to the SUS scores, the usability of the ReHapticKnob was rated between good and excellent [44, 45]. The mean SUS score (> 79) is slightly higher than the one reported by other groups using rehabilitation technologies with minimal or no supervision, as for example the ironHand glove (mean SUS = 73 [9]) or the unactuated device MERLIN (mean SUS = 71.94 and 77.27 in [46] and [47], respectively). The SUS score remained in the same range as the one found in a previous usability study where we simulated one minimally supervised therapy session with the ReHapticKnob [12], suggesting that the changes made to the device and the prolonged interaction with it did not negatively impact usability.

Regarding the NPS, although the score obtained is not high (27%), of the 11 participants considered for this analysis, the majority were promoters (6 and 5 for Usability 1 and 2, respectively) or passively satisfied participants (2 and 4), while the detractors were fewer (3 and 2). This, combined with a high customer satisfaction score, points to a good user experience, although the reasons why some users were dissatisfied should be examined further.

The checklist performed at the end of the minimally supervised weeks did not identify recurrent issues for the device. To be more efficient in the everyday clinical practice, the checklist could be updated at every supervised or minimally supervised session instead of after two weeks only, allowing for less supervised sessions and a faster transition to unsupervised therapy for patients who can quickly learn how to use the device.

### Limitations and future work

This study included a relatively small sample size, potentially affecting statistical power. However, this sample size allows to draw important first conclusions on feasibility of unsupervised therapy with the ReHapticKnob and to pave the way for subsequent clinical studies in home settings. Indeed, while in this study the device was used without any supervision in a clinical setting, the structured environment of the clinic and the routine to which inpatients are used to may have influenced motivation to train and led to a higher dose of unsupervised robot-assisted therapy than what could be expected in a home environment. At the same time, the already busy therapy schedule may also have negatively affected the dose of unsupervised robotic therapy that could be achieved due to fatigue or simply due to a lack of available time. Evaluating unsupervised robot-assisted therapy with the ReHapticKnob in the home of stroke patients will therefore be the next necessary step, and a follow-up clinical study with a portable version of the ReHapticKnob is planned.

## Conclusion

We could successfully demonstrate that after a supervised familiarization phase, unsupervised upper limb robot-assisted therapy in a clinical setting with an actuated end-effector device was feasible and can lead to a meaningful increase in therapy dose. Results concerning usability show that the ReHapticKnob is well accepted and well usable also without supervision by patients with no severe cognitive deficits. The results presented here support the use of the ReHapticKnob for unsupervised therapy as a complement to usual care in clinical settings and pave the way to its application in home settings.

### Electronic supplementary material

Below is the link to the electronic supplementary material.

Additional File 1

Additional File 2

## Data Availability

The datasets generated and analysed during the current study are available from the corresponding author upon reasonable request.

## Abbreviations

BBT: Box and Block Test
CSAT: Customer Satisfaction Score
FMA-UE: Fugl-Meyer Assessment of Upper Extremities
LCF-R: Levels of Cognitive Functioning-Revised
mAS: modified Ashworth Scale
MESUPES: Motor Evaluation Scale for Upper Extremities in Stroke Patients
NIHSS: National Institutes of Health Stroke Scale
NPS: Net Promoter Score
PSSUQ: Post-Study System Usability Questionnaire
SUS: System Usability Scale
TLX: raw Task Load Index
VASp: Visual Analogue Scale for pain
