# Supplementary figures and images for: Unsupervised robot-assisted rehabilitation after stroke: feasibility, effect on therapy dose, and user experience
Source: J Neuroeng Rehabil. 2024 Apr 9;21:52. doi: 10.1186/s12984-024-01347-4 (PMC11005116; doi:10.1186/s12984-024-01347-4)

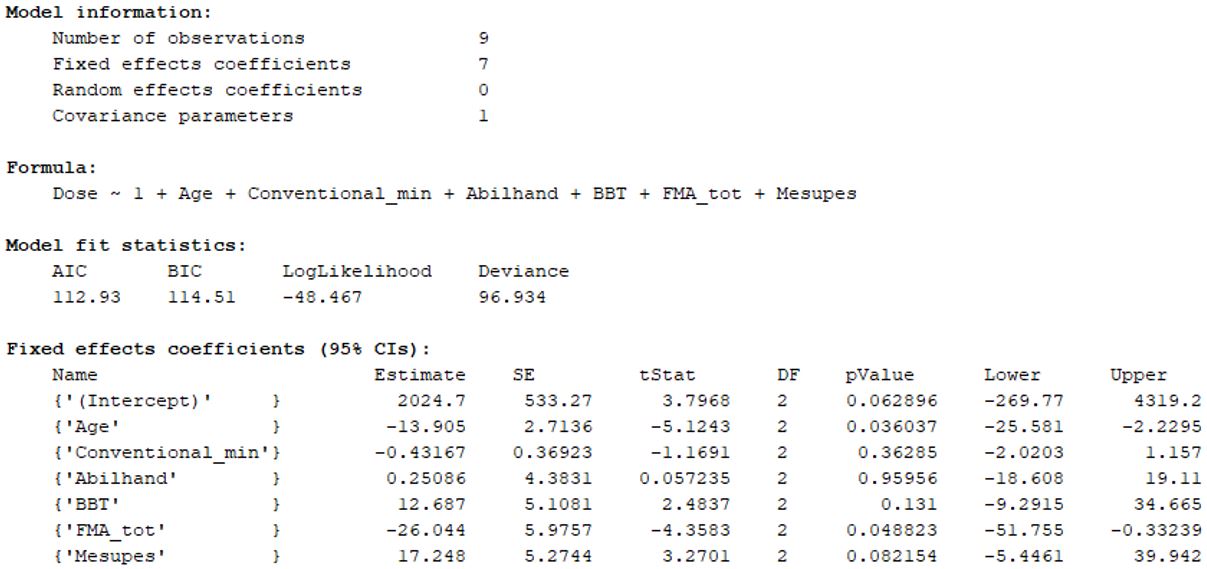

Supplement: Supplementary file 1 — Additional File 1 [file 12984_2024_1347_MOESM1_ESM.jpg]

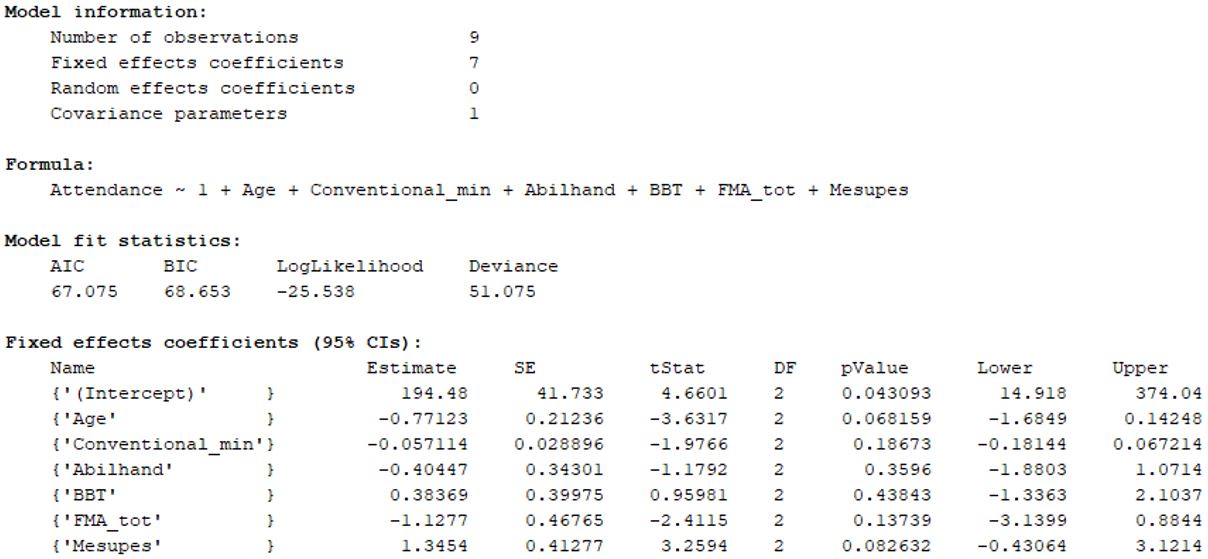

Supplement: Supplementary file 2 — Additional File 2 [file 12984_2024_1347_MOESM2_ESM.jpg]
